# Ophthalmic services utilisation and associated factors in the Ashanti region, Ghana

**DOI:** 10.4314/gmj.v57i1.9

**Published:** 2023-01

**Authors:** Abdul-Kabir Mohammed, Alvin J Munsamy

**Affiliations:** 1 Discipline of Optometry, College of Health Sciences, University of KwaZulu-Natal, Durban, South Africa; 2 Department of Optometry and Visual Science, Kwame Nkrumah University of Science and Technology, Kumasi, Ghana

**Keywords:** Ophthalmic services, Visual impairment, Ashanti region, Ghana

## Abstract

**Objective:**

This survey determined the utilisation of eye care services and associated factors among adults in the Ashanti region of Ghana.

**Design:**

A population-based cross-sectional descriptive study

**Method:**

Data for this study was collected from 1615 randomly selected individuals in the Ashanti region of Ghana, using a structured, pretested interviewer-guided questionnaire. Information regarding the accessibility and determinants of, and barriers to, eye care services was based on self-reports, using the WHO Eye Care Services Assessment Questionnaire. Inferential analyses were performed using the chi-square test for statistical significance, set at p=0.05.

**Setting:**

Ashanti Region, Ghana

**Participants:**

One thousand six hundred and fifteen randomly selected adults

**Results:**

Public eye care facilities were used by 58.2% of the participants for their last eye exam. Of the participants, 47.0% had travelled less than five kilometres for their last eye exam. Waiting time and service cost were participants' most frequently cited challenges in seeking care. No need felt (40.1%), self-medication (37.7%) and cost (22.2%) were the most frequently mentioned barriers to seeking ophthalmic services.

**Conclusion:**

The major challenges encountered in seeking eye care services were waiting time and cost of service. Major barriers to ophthalmic services utilisation were no need felt, self-medication and cost. Factors such as cost, lack of felt need and self-medication, which serve as barriers to utilising eye care services, should be addressed by stake-holders through eye health education and promotion.

**Funding:**

None declared

## Introduction

Blindness is one of the most tragic, but sometimes avoidable, disabilities, especially in the least developed countries.^1^ Given this, the World Health Organization (WHO) and the International Agency for the Prevention of Blindness (IAPB) initiated the ‘VISION 2020: the right to sight’ campaign to prevent 100 million cases of avoidable blindness by the year 2020.^2^

The global burden of visual impairment and avoidable blindness is increasing.^3^ The preventable causes of visual impairment and blindness account for up to 80% of the global burden.^4^ The least developed countries carry the greatest burden of visual impairment, with the majority of the global burden found in India and sub-Saharan Africa.^5^

Cross-sectional studies conducted in developing countries have generally reported low eye care service utilisation levels.^6^,^7^ Ophthalmic service utilisation in some developed countries has also been found to be less than ideal.^8^ Non-affordability and poor accessibility of the services have been identified as important reasons for the high prevalence of avoidable, blinding eye diseases.^9^ According to Di Stefano^10^, the lack of accessible and affordable eye and vision care globally is a critical barrier to successfully eliminating avoidable blindness.

Payment for health care services in Ghana is through cash or the National Health Insurance Scheme (NHIS). Under the NHIS, operated by the government, citizens who pay an annually renewable subscription fee, and elderly persons (70 years and above), receive free, selected medical services covered by the scheme, including some ophthalmic services.^11^

Payment for health care at private health institutions is mostly by cash, and only a small portion of the population can afford private health care.

Records indicate that over half the people signed up for the NHIS are the rich; rather than the poor and vulnerable, for whom it is primarily intended.^12^ The government of Ghana puts the coverage rate of the NHIS at about 70% of the population, but the actual coverage could be as low as 18% of the poor compared to 64% of the wealthy.^13^

In Ghana, the introduction of the NHIS in 2003 generally improved access to health care.^14^ Eye care services covered by the NHIS include consultation, cataract surgery and refraction fees in public and private health facilities accredited by the National Health Insurance Authority (NHIA).^14^Eye care in lower-income countries, including Ghana, is affected by an undesirable and inequitable distribution of personnel.^15^ A recent study to assess the progress in achieving the ‘Vision 2020: right to sight initiative’ in Ghana reported an inadequate and uneven distribution of eye care human resources in Ghana.^16^ It showed that routine eye care services were unavailable at sub-district and community-level facilities, making up 90% of all health facilities nationwide.^16^

Access to affordable eye care services is an important determinant of eye care utilisation, a primary factor in preventing avoidable visual impairment and blindness. This study aims to determine the utilisation of ophthalmic services and related factors among adults in the Ashanti region of Ghana. The Ashanti region was chosen for this study because of its strategic location in Ghana. The region is centrally located in the middle belt of Ghana and shares boundaries with six of the sixteen regions of Ghana. Data from the region may, within reason, help inform the state of access to eye care services in the entire country.

## Methods

### Data and Sampling

The study used a population-based, cross-sectional, descriptive design that employed a multistage sampling technique. Inclusion criteria included: being 18 years and over, residing in the Ashanti region of Ghana, and voluntarily consenting to participate in the study. A probability technique using proportion-to-population size was employed to randomly select 50 electoral areas (the number of electoral areas selected was dependent on the district population) from 10 of the 43 districts in the Ashanti region of Ghana. After that, 15 households were selected per electoral area, which comprised 750 households. Within the households, 1,804 individuals met the inclusion criteria, of which 1615 (89%) agreed to be interviewed. All participants were residents of the 10 districts randomly selected between January 2021 and June 2021.

### Data Collection

A structured interviewer-guided questionnaire was used to obtain respondents' socio-demographic characteristics and information regarding accessibility, utilisation, and barriers to eye care services. This information was based on self-reports, using items derived from the WHO Eye Care Services Assessment Questionnaire,^17^ with minor modifications. The questionnaire consisted of two sections. Section One sought information on the socio-demographic characteristics of the respondents, including gender, age, level of education and approximate monthly income. Section Two focused on whether the participants had ever had an eye examination; their reasons for their last eye examination; the challenges encountered when seeking an eye examination; and barriers to eye care services utilisation.

All interviews were conducted by trained field workers who were all teaching and research assistants in the Department of Optometry at the Kwame Nkrumah University of Science and Technology, Kumasi, Ghana.

### Ethical Considerations

Ethical approval for the study was obtained from the Biomedical Research Ethics Committee (BREC) of the University of KwaZulu-Natal, South Africa (Ref: BREC/00001787/2020) and the Committee on Human Research, Publications and Ethics of the Kwame Nkrumah University of Science and Technology, Kumasi, Ghana (Ref: CHRPE/AP/006/17). Gatekeeper consent was obtained from the Ashanti Regional Health Directorate. Informed written consent was obtained from all the survey participants. All study procedures adhered to the tenets of the Declaration of Helsinki.

### Data Analysis

The data was entered into a Microsoft Excel worksheet (Microsoft Inc., USA), and was cleaned, coded and exported into the Statistical Package for Social Sciences Software, Version 25 (SPSS 25). The data was then analysed using descriptive statistics. Frequencies and percentages were used to summarise the data, after which inferential analyses were performed using the chi-square test for statistical significance, which was set at p=0.05. Results were presented in the form of tables.

## Results

A total of 1,615 of the 1,804 eligible individuals participated in this study, giving a response rate of 89%. The participants' mean age and standard deviation were 36.2+/-15.5 years, with an age range of 18-82 years.

Of the sample, 54.4% were females, and 52.0% lived in urban districts. The 18-29-year age group was the largest (48.7% of the respondents), followed by the 30-34-year age group (26.7%).

The majority of the respondents (87.7%) had some form of secular education, and 25.6% had up to tertiary education; 68.4% reported that they were in employment; 27.1% of respondents earned less than or equal to 500 cedis (83 US dollars, the lowest wealth index) monthly; whilst only 0.4% earned more than 5000 cedis (826 US dollars, the wealthiest index). In addition, 12.3% reported being diagnosed with one or more systemic diseases: 3.9% were diabetic, 9.8% were hypertensive, and 0.3% had sickle cell disease.

Furthermore, 34.5% reported a change in their vision within the last two years; 85.3% of the participants reported that they felt regular eye examinations were necessary, even without symptoms; and 87.3% felt children under five required eye examinations.

### Factor Comparisons by Gender

Table 1 presents the predisposing, enabling and need factors of the participants, according to gender.

**Table 1:** Predisposing, enabling and need factors; comparisons by gender

Variable	Total (N =1615)	Males (N =737, 45.6%)	Females (N =878, 54.4%)	
	n (%)	n (%)	n (%)	*p*-value*
**Predisposing factors**				
***Age (mean, SD)***	36.2 (15.6)	35.1 (14.7)	37.2 (16.2)	**<0.001**
**Age groups (years)**				
**18–29**	787 (48.7)	370 (50.2)	417 (47.5)	0.094
**30 – 44**	413 (26.7)	204 (27.7)	227 (25.9)	
**45 – 59**	227 (14.1)	102 (13.8)	125 (14.2)	
**60 – 74**	127 (7.9)	47 (6.4)	80 (9.1)	
**≥75**	43 (2.7)	14 (1.9)	29 (3.3)	
**Enabling factors**				
***District***				
**Amansie South**	129 (8.0)	53 (7.2)	76 (8.7)	**<0.001**
**Asokore Mampong**	279 (17.3)	134 (18.2)	145 (16.5)	
**Asokwa**	215 (13.3)	97 (13.2)	118 (13.4)	
**Atwima Nwabiagya**	182 (11.3)	89 (12.1)	93 (10.6)	
**Bosomtwe**	186 (11.5)	62 (8.4)	124 (14.1)	
**Ejisu**	151 (9.3)	75 (10.2)	76 (8.7)	
**Kumawu**	101 (6.3)	34 (4.6)	67 (7.6)	
**Amansie West**	116 (7.2)	41 (5.6)	75 (8.5)	
**Offinso North**	60 (3.7)	27 (3.7)	33 (3.8)	
**Old Tafo**	196 (12.1)	125 (17.0)	71 (8.1)	
***Level of education***				
**None**	199 (12.3)	62 (8.4)	137 (15.6)	**<0.001**
**Primary**	124 (7.7)	29 (3.9)	95 (10.8)	
**Intermediate**	449 (27.8)	172 (23.3)	227 (31.5)	
**Secondary**	429 (26.6)	215 (29.2)	214 (24.4)	
**Tertiary**	414 (25.6)	259 (35.1)	155 (17.7)	
***Employed***	1103 (68.3)	527 (71.5)	576 (65.6)	**0.011**
***Monthly income (cedis)***				
**≤ 500**	439 (27.1)	164 (22.3)	274 (31.2)	**<0.001**
**501 – 1000**	364 (22.5)	182 (24.7)	182 (20.7)	
**1001 – 2000**	220 (13.6)	117 (15.9)	103 (11.7)	
**2001–4999**	75 (4.6)	59 (8.0)	16 (1.8)	
**≥.5000**	6 (0.4)	5 (0.7)	1 (0.1)	
**Needs factors**				
***Presence of systemic disease***				
**Diabetes**	63 (3.9)	17 (2.3)	46 (5.2)	0.488
**Hypertension**	159 (9.8)	52 (7.1)	107 (12.2)	0.138
**Sickle cell disease**	5 (0.3)	0 (0.0)	4 (0.5)	0.135
***Observed change in vision in the last*** ***two years***	557 (34.5)	228 (30.9)	329 (37.5)	**0.006**
***Think children under five need eye*** ***examinations***	1410 (87.3)	639 (86.7)	771 (87.8)	0.504
***Think regular eye examinations are*** ***important***	1377 (85.3)	655 (88.9)	722 (82.2)	**<0.001**
***Visit eye clinic any time they have an*** ***eye problem***	279 (17.3)	126 (17.1)	153 (17.4)	0.984
***Period since last eye examination***				
**Less than a year**	211 (13.0)	87 (11.8)	124 (14.1)	**<0.001**
**1 to 2 years**	194 (12.0)	110 (14.9)	84 (9.6)	
**2 to 3 years**	96 (5.9)	57 (7.7)	39 (4.4)	
**3 to 5 years**	85 (5.3)	33 (4.5)	52 (5.9)	
**More than 5 years**	82 (5.1)	39 (5.3)	43 (4.9)	
**Don't remember**	23 (1.4)	7 (0.9)	16 (1.8)	

*Predisposing Factors*

There was a significant gender difference in the mean age of the participants (males: 35.1; females: 37.2, p < 0.001). However, there was no significant gender difference in terms of the age groups of the participants (p = 0.094).

*Enabling Factors*

Table 1 also shows that a significantly higher proportion of females did not have any form of education (males: 8.4%; females: 15.6%). In addition, significantly more males had secondary (males: 29.2%; females: 24.4%) and tertiary (males: 35.1%; females: 17.7%) education (p<0.001). Table 1 goes on to show significant gender differences in constituency (p < 0.001), employment status (p = 0.011) and monthly income (p < 0.001).

*Need Factors*

The result indicated significant gender differences in the participants with regards to vision problems (p = 0.006); participants who thought regular eye examinations were important (p < 0.001); and the period since the last eye examination (p = 0.001). However, there was no significant gender difference in participants' systemic health, which included diabetes (p = 0.488), hypertension (p = 0.138) and sickle cell anaemia (p = 0.135).

### Factors related to eye care utilisation and accessibility

Of the 1615 participants, only 691 (42.8%) had ever had an eye examination. Of those who had previous eye examination, 333 (48.2%) were males, while 358 (51.8%) were females. Table 2 presents the distribution of factors related to eye care utilisation and accessibility for the respondents.

**Table 2:** Factors related to eye care utilisation and accessibility

Accessed variable	Total (N = 691, 42.8)	Males (N =333, 48.2%)	Females (N =358, 51.8%)	p-value*
	n (%)	n (%)	n (%)
**Facility type for last eye examination**				
**Public eye clinic**	402 (58.2)	161 (48.3)	241 (67.3)	**<0.001**
**Private eye clinic**	130 (18.8)	75 (22.5)	5 (15.4)	
**Community-based Health Planning and Services** **Centre (CHPS)**	6 (0.9)	4 (1.2)	2 (0.6)	
**DVLA**	42 (6.1)	38 (11.4)	4 (1.1)	
**During eye screening**	67 (9.7)	32 (9.6)	35 (9.8)	
**Others**	3 (0.4)	0 (0.0)	3 (0.8)	
**Not specified**	41 (5.9)	23 (6.9)	18 (5.0)	
**Distance travel to facility (kilometres)**				
**Less than 5**	326 (47.0)	137 (41.0)	189 (52.6)	0.065
**5 – 10**	253 (36.5)	125 (37.4)	128 (35.7)	
**More than 10**	54 (7.8)	31 (9,3)	23 (6.4)	
**Time spent at the facility (hours)**				
**Less than an hour**	272 (39.2)	125 (37.4)	147 (40.9)	0.499
**1 – 2**	252 (36.4)	114 (34.1)	138(38,4)	
**2 – 4**	86 (12.4)	41(12.3)	45(12.5)	
**More than 4**	32 (4.6)	19(5.7)	13(3.6)	
**Time taken to the facility (hours)**				
**Less than an hour**	462 (66.7)	210 (62.9)	252 (70.2)	0.436
**1 – 2**	155 (22.4)	75 (22.5)	80 (22.3)	
**2 – 4**	24 (3.4)	11 (3.2)	13 (3.6)	
**More than 4**	2 (0.5)	2 (0.3)	0 (0.0)	
**Satisfied with care given**	598 (86.3)	271 (81.1)	327 (91.1)	**0.002**
**Rating of eye care services**				
**Very poor**	6 (0.9)	4 (1.2)	2 (0.6)	**0.001**
**Poor**	34 (4.9)	28 (8.4)	6 (1.7)	
**Good**	222 (32.0)	104 (31.1)	118 (32.9)	
**Satisfactory**	131 (18.9)	56 (16.8)	75 (20.9)	
**Very good**	168 (24.2)	73 (21.9)	95 (26.5)	
**Excellent**	74 (10.7)	30 (9.0)	44 (12.3)	
**Reason For Last Eye Examination**				
**Routine eye exams**	152 (21.9)	92 (27.5)	60 (16.7)	**<0.001**
**Distant vision difficulty**	222 (32.0)	106 (31.7)	116 (32.3)	0.918
**Near vision difficulty**	2 (0.3)	0 (0.0)	2 (0.6)	0.173
**Pain**	132 (19.0)	39 (11.7)	93 (25.9)	**<0.001**
**Tearing**	119 (17.2)	36 (10.8)	83 (23.1)	**<0.001**
**Discharge**	33 (4.8)	8 (2.4)	25 (7.0)	**0.005**
**Headache**	8 (1.2)	5 (1.5)	3 (0.8)	0.410
**Itchy eyes**	105 (15.2)	32 (9.3)	73 (20.3)	**<0.001**
**Acquiring driver's license**	40 (5.8)	36 (10.8)	4 (1.1)	**<0.001**
**Others**	62 (8.9)	34 (10.2)	28 (7.8)	0.260

Public eye care facilities were used by 58.2% of the participants for their last eye examination. Only 18.0% utilised private facilities. Significantly more females utilised public facilities compared to males (67.3% vs. 48.3%, p<0.001). Males were found to be significantly more likely to have utilised private facilities, compared to females (22.5% versus 15.4%, p<0.001). A significantly higher proportion of males (11.4%) had their last eye examination at the premises of the Driver and Vehicle Licensing Authority (DVLA) as a requirement for their drivers' license renewal, compared to females (1.1%), p<0.001. Almost one in ten (9.7%) of the participants had their last eye examination during eye screening exercises.

The results of the study indicated that only 47% of the participants had travelled less than five kilometres to seek eye care and their last eye examination; and fewer than two-out-of-five (39.2%) spent less than an hour at the facility. There was no significant gender difference in distance travelled to seek eye care and time spent at the facility (p=0.065 and p=0.499, respectively). More than two thirds (66.7%) accessed the facility in less than an hour and 0.2% reported taking more than four hours to reach the facility.

The majority (86.3%) of the participants reported being satisfied with the care they received during their last eye examination. Only 0.9% and 4.9% of the respondents rated the care received as very poor and poor, respectively. The other ratings for the care received by the participants in their last eye examinations were good (32.0%); satisfactory (18.9%); very good (24.2%); and excellent (10.7%). Females were significantly more satisfied with the care received, compared to males (p=0.002).

The result further shows that the major reasons cited for seeking eye care were difficulty with distance vision (32.0%) and for a routine eye examination (21.9%). Difficulty with near vision (0.3%) was the reason least-cited by the participants for seeking their last eye examinations.

### Challenges encountered during last eye examination

Among the 691 respondents who had ever had an eye exam, 249 (36%) reported encountering challenges in seeking care at their last eye examination. Table 3 shows the distribution of reported challenges encountered in seeking eye care.

**Table 3:** Challenges encountered in seeking eye care

Variables	Distance to facility	Cost	Self-medication	Reputation of the facility	Use of traditional means	No need felt	Time	N
	n (%)	n (%)	n (%)	n (%)	n (%)	n (%)	n (%)	
**Gender**								
Male	26 (5.7)	82 (17.9)	161 (35.1)	1 (0.2)	8 (1.7)	171 (37.3)	21 (4.6)	**459**
Female	23 (4.1)	143 (25.8)	221 (39.8)	4 (0.7)	5 (0.9)	236 (42.5)	28 (5.0)	**555**
	*p = 0.261*	***p = 0.003***	*p = 0.121*	*p = 0.255*	*p = 0.235*	*p = 0.089*	*p = 0.728*	
**Age Group**								
18 – 29	26 (5.3)	105 (21.2)	170 (34.3)	4 (0.8)	3 (0.6)	204 (41.2)	32 (6.5)	**495**
30 – 44	11 (3.9)	45 (16.1)	102 (36.6)	1 (0.4)	1 (0.4)	126 (45.2)	8 (2.9)	**279**
45 – 59	5 (3.8)	33 (25.4)	67 (51.5)	0 (0.0)	1 (0.8)	38 (29.2)	7 (5.4)	**130**
60 – 74	3 (3.8)	24 (30.0)	32 (40.0)	0 (0.0)	7 (8.8)	32 (40.0)	2 (2.5)	**80**
≥ 75	4 (13.3)	18 (60.0)	11 (36.7)	0 (0.0)	1 (3.3)	7 (23.3)	0 (0.0)	**30**
	*p = 0.210*	***p < 0.001***	***p < 0.001***	*p = 0.682*	***p < 0.001***	***p = 0.011***	***p = 0.101***	
**Level of education**								
None	6 (4.9)	52 (42.3)	75 (61.0)	0 (0.0)	1 (0.8)	20 (16.3)	1 (0.8)	**123**
Primary	3 (3.8)	35 (44.3)	34 (43.0)	0 (0.0)	0 (0.0)	26 (32.9)	0 (0.0)	**79**
Intermediate	17 (5.9)	84 (29.2)	102 (35.4)	0 (0.0)	8 (2.8)	133 (46.2)	10 (3.5)	**288**
Secondary	6 (1.9)	33 (10.6)	109 (35.2)	4 (1.3)	4 (1.3)	145 (46.8)	25 (8.1)	**310**
Tertiary	17 (7.9)	21 (9.8)	62 (39.0)	1 (0.5)	0 (0.0)	83 (38.8)	13 (6.1)	**214**
	***p = 0.026***	***p < 0.001***	***p < 0.001***	*p = 0.168*	*p = 0.058*	***p < 0.001***	***p < 0.001***	
**Monthly income (cedis)**								
Not employed	20 (5.8)	86 (25.1)	100 (29.2)	3 (0.9)	8 (2.3)	147 (43.0)	11 (3.2)	**342**
≤ 500	7 (2.6)	73 (26.6)	116 (42.3)	1 (0.4)	0 (0.0)	105 (38.3)	12 (4.4)	**274**
501 – 1000	14 (6.3)	57 (25.4)	85 (37.9)	0 (0.0)	1 (0.4)	92 (41.1)	10 (4.5)	**224**
1001 – 2000	5 (4.1)	9 (7.4)	51 (41.8)	0 (0.0)	4 (3.3)	47 (38.5)	14 (11.5)	**122**
2001–4999	3 (6.3)	0 (0.0)	29 (60.4)	1 (2.1)	0 (0.0)	13 (27.1)	2 (4.2)	**48**
≥5000	0 (0.0)	0 (0.0)	1 (0.4)	0 (0.0)	0 (0.0)	3 (75.0)	0 (0.0)	**4**
	*p = 0.370*	***p < 0.001***	***p < 0.001***	*p = 0.377*	***p = 0.031***	*p = 0.208*	*p* = **0.015**	

N, total sample per category; n, frequency; %, percentage frequency; *p*, Pearson Chi-Square

Table 3 shows that the major challenges reported by the participants when seeking eye care were waiting time (males: 78.0%; females: 62.7%) and cost (males: 39.8%; females 36.5%). Significantly more males than females reported waiting time (males: 78.0%; females: 62.7%, p =0.008) as a challenge encountered in seeking care, whereas caregiver attitude (p=0.008) and the quality of care (p =0.005) were significantly more important for the females than the males.

There was a significant age variation in participants' report of waiting time (p <0.001) and quality of service (p <0.001) as challenges encountered when seeking eye care. Level of education significantly varied among the subjects citing waiting time (p = 0.004); cost of service (p =0.007); and quality of service (p <0.001), as challenges encountered when seeking eye care. The monthly income of the respondents was found to only be significantly associated with cost (p =0.027) of service (see Table 3).

## Discussion

The study evaluates access to the services for adults, and barriers to their utilisation, in the Ashanti region of Ghana. Our study showed that most (58.2%) participants had their last eye examination in a public eye care facility, whilst 18.0% utilised private facilities for their last eye examination. The choice of public-funded eye clinics, rather than private, by the majority of the respondents, may be due to the direct cost of the service, which is cheaper at the public facilities than the private ones. In addition, the NHIS coverage renders public facilities more affordable. Private eye care services are more costly compared to public services and are often beyond the means of the poor in most lower-income countries.^16^

Only 42.8% of the respondents had ever had their eyes examined, which is cause for concern. Less than two out of five (39.2%) of those who had ever had their eyes examined travelled less than five kilometres to seek eye care, which raised proximity to the nearest eye care facility as an important factor in providing accessible eye care services. The cost of transportation, related to proximity, has the potential to be a barrier to seeking eye care at these facilities.

Studies in Ghana^18^, Nigeria^19^ and Fiji^20^ have found that the cost of transport and distance to eye care facilities are barriers to eye care utilisation.

Proximity is not limited to developing countries: O'Connor et al.^21^ listed proximity and convenience as main facilitators of eye care services use in Australia, citing transport and the need for an accompanying person as barriers to utilising low-vision services. Wang et al.^22^ also found that limited access was a barrier to treating glaucoma among African American Medicare beneficiaries. The satisfaction expressed with the care received during the last eye examination was remarkable (86%) among the participants, which is a good outcome, as a happy patient is likely to recommend the use of eye care facilities to other people and is also more likely to comply with treatment. This finding may indicate that the expectations of most patients were met at the ophthalmic facilities visited. The result compares with that of a study in the Upper East region of Ghana,^23^ which reported overall satisfaction of 90% among patients attending the eye clinic. Studies in Uganda^24^, Nepal^25^, Central India^26^ and Brazil^27^ showed overall satisfaction of 79%, 74%, 78%, and 77%, respectively, all relatively lower than in this study. The varying levels of satisfaction may be due to the different ways the services were measured, study population or socio-cultural differences.

Waiting time and service cost were participants' most frequently cited challenges in seeking care. This is in agreement with studies conducted in the central region of Ghana 15 and Edo State, Nigeria^19^, which also found cost and waiting time to be the major challenges faced in seeking eye care services. Ndegwa et al.^28^ and Elam and Lee^29^ also reported a lack of finance as one of the main challenges faced in seeking eye care in Kenya. In many rural areas and some urban centres in Africa, poverty is a major issue, and for that reason, the cost of eye care services and transportation costs to eye care facilities put eye care beyond the reach of the underprivileged. Hence, conditions which could have been treated when timeously presented to eye care facilities may result in avoidable visual impairment and blindness.

The lack of felt need, self-medication and cost were the main barriers to using eye care services among the respondents. No perceived need to seek eye care is concerning, as this may result from trivialising the signs and symptoms of eye conditions. Visual problems can be asymptomatic in the initial stages, and delays in seeking treatment can result in irreversible vision loss. This is consistent with other studies that reported a lack of felt need as a reason for not seeking eye care.^19^,^18^

Self-medication as a barrier to seeking professional ophthalmic care was common in other studies, especially in developing countries such as Malawi.^30^ Nigeria^31^ and Egypt.^32^ Although this study did not investigate the reasons for self-medication among the respondents, it can be ascribed to challenges of accessibility, availability and affordability of the ophthalmic services, as supported by evidence from several studies.^9^,^10^,^33^,^34^ Cost as a barrier has also been found in other studies in Ghana^15^,^18^ and other countries.^7^,^28^,^35^

A major limitation of the study was the use of 10 of the 43 districts in the region due to logistical constraints and having to observe COVID-19 protocols throughout the data collection period. Another limitation is the exclusion of persons below 18 years, which might have affected the study outcomes, as paediatric eye care services are sometimes inadequate in developing countries regarding facilities, equipment and skilled professionals. Also, the factors analysed in this study were assessed through self-reporting and were subject to recall bias and the possible effects of social desirability.

The GHS (Ghana Health Services) should mandate and resource the National Eye Care Unit (NECU) to promote and integrate eye health at all levels of healthcare delivery. The government of Ghana and the various stakeholders in eye care in Ghana need to focus on strategies to address factors such as cost and waiting time, which serve as barriers to utilising eye care services. Lack of felt need and self-medication, as barriers, should also be addressed through eye health education and promotion, emphasising the benefits of routine eye examinations and the likelihood of preventing visual impairment and blindness when eye problems are presented for timely management.

## Conclusion

This study revealed inadequate access to eye care services as a significant barrier to eye care utilisation in the region concerned. The major challenges encountered in seeking eye care were waiting time and cost of service. Major barriers to ophthalmic services utilisation were the lack of felt need, self-medication and cost. The GHS need to establish programmes that would make eye care accessible to the underserved, thereby minimising the unmet need for the services in the region. Implementing preventive measures, such as monthly or quarterly vision screening at the sub-district level, may improve access to eye care at the district level.
